# Insights and Advancements in Biomaterials for Prosthodontics and Implant Dentistry

**DOI:** 10.3390/molecules27165116

**Published:** 2022-08-11

**Authors:** Artak Heboyan, Muhammad Sohail Zafar, Dinesh Rokaya, Zohaib Khurshid

**Affiliations:** 1 Department of Prosthodontics, Faculty of Stomatology, Yerevan State Medical University after Mkhitar Heratsi, Str. Koryun 2, Yerevan 0025, Armenia; 2 Department of Restorative Dentistry, College of Dentistry, Taibah University, Al Madinah Al Munawwarah 41311, Saudi Arabia; 3 Department of Dental Materials, Islamic International Dental College, Riphah International University, Islamabad 44000, Pakistan; 4 Department of Clinical Dentistry, Walailak University International College of Dentistry, Walailak University, Bangkok 10400, Thailand; 5 Department of Prosthodontics and Implantology, College of Dentistry, King Faisal University, Al-Ahsa 31982, Saudi Arabia

It is always difficult to avoid subjectivism in the assessment of the properties of prosthodontics materials due to the ambiguity in the evaluation criteria of dental research. However, over the past five years, the interest in this field of dentistry and its development in research has been increasing. As a result, a large number of new biomaterials have been introduced in dentistry. Those worth mentioning include titanium (Ti) and Ti alloys, yttria-stabilized zirconia (YTZP), zirconium-reinforced lithium silicate, lithium-disilicate-reinforced glass ceramics, as well as acrylic resins with enhanced mechanical properties. In addition to these new materials, glass fibers and nanoparticles present high potential in the reinforcement of other base materials and resinous structures. Remarkably, the improvement of the mechanical features of polymethyl methacrylate (PMMA) denture base materials can be achieved by using a hybrid system, hybrid fillers or their combination as a new reinforcement system. Research in this area is typically performed in vitro, while new approaches using in vivo methods are currently in demand [1,2,3,4,5,6,7].

Due to their excellent translucency and esthetic properties, newly developed multi-color monolithic ceramic materials are highly durable and can be recommended for use in applications in which strength is required, and these materials are also associated with aesthetic improvements. Therefore, the increasing prevalence of conservative treatment using adhesively luted monolithic restorations is justified. In this Special Issue, we expected positive results to be published based on previous studies on ceramic restauration, especially in terms of implant-supported single crowns and FDPs [1]. 

In addition to a material’s properties, the stress concentration of a material is an important factor affected by the type of marginal preparation, endodontic treatment, and superstructure used. In terms of stress magnitude, contemporary concepts such as the biologically oriented preparation technique (BOPT) can be a promising choice for anterior monolithic zirconium crowns, despite the fact that the highest stress magnitude exists at the restoration margin [2]. From the biological point of view, according to recent cytomorphometric, bacteriological, and hygienic-based investigations, zirconium restorations showed better results compared with conventional Co-Cr-based ceramic restorations [3,4,5,6,7].

According to the literature, ceramic surface conditioning is negatively influenced by a number of erosive factors, such as the low pH of most beverages. This fact should not be neglected, and patients who undergo prosthetic treatment should be warned of the effects of corrosion. A longer period of storage was also noted as a factor which may lead to an increase in roughness on the surface of ceramics [8]. 

Relevant fields of study to the investigation of the factors that cause the deterioration of retentive potential of materials are thermal undulation and water dispersion. Thus, retention loss was found to be higher in nylon and polyetheretherketone (PEEK) (which contain strong polar groups in polymeric chains) than in polyvinyl siloxane (PVS). However, all of the polymeric materials evaluated showed a decrease in retention after one year of artificial aging. Therefore, more investigations regarding attachment systems suitable for the repair of abutments in vulnerable patients are still necessary [9,10,11].

The use of an adhesive system allows the creation of a solid bond between copings and resin cement; resin cement should not be applied on its own. An adhesive should be applied in clinical practice to achieve better outcomes. Therefore, tensile bond strength investigations [12] should be performed considering different materials that share the same indication.

At present, zirconium is the most frequently used restorative material, being the color of natural teeth, while PEEK can be used as an alternative in more flexible superstructures. Zirconium crowns were demonstrated to withstand three times more antagonist wear and had higher color stability. These crowns exhibit the least displacement, as opposed to crowns made of PEEK. However, PEEK prosthetic crowns demonstrate minimal abrasion, better stress modulation due to plastic deformation, as well as enhanced color stability, which means they could be an alternative to zirconium in crown fabrication. The choice of material used in the manufacture of dentures depends on the purpose of use and, accordingly, takes into account its properties, advantages, and limitations [13].

The characteristics of PMMA make it the perfect base material for prostheses. This material can be used in denture rebases, reliners, maxillofacial prostheses, orthodontic appliances, temporary restorations, and even in splints for surgical procedures. Qualities that make denture base materials widely used are esthetics, precise fabrication, and an easy manufacturing technique. They should also be repairable, available, and inexpensive. In spite of many positive features, PMMA still has some disadvantages. A major shortcoming of PMMA is its insufficient toughness, resulting in the frequent need for repair during the span of a year. Another widespread problem is that some individuals exhibit allergic reactions to acrylic resin, which can be solved by modifying resin prosthesis bases [9,10,11,12,13].

The use of nanoparticles as reinforcements in conventional glass ionomer cement is another field that requires attention. A previous investigation revealed that the 8% addition of a titanium reinforcement in glass ionomer cement decreases the wear rate from almost 35 to 25%. The surface hardness, however, was not enhanced. Among the factors which may have an impact upon the mechanical characteristics of materials are the environment, mixing time, and proportion of resin [14]. Research into these parameters could contribute to the scientific literature and improve the field of dentistry. 

As an oral implant material, titanium alloy has a minor drawback: its relatively low wear resistance. Nanostructured ceramic coatings such as TiN, ZrO_2_/SiO_2_, Si_3_N_4_/TiO_2_, and ZrO_2_/Al_2_O_3_ are currently used to solve this problem. Due to its improved adhesion power and lower porosity, a bilayered coating demonstrated a 200- and 500-fold increase in wear resistance, as opposed to the monolayer Al_2_O_3_-13TiO_2_ and ZrO_2_, respectively [1]. New coating layers should be developed and applied in clinical scenarios. 

Different methods were applied by researchers to manufacture metal surface nano-crystallization in order to improve the biological activity of a metal. A nano-textured titanium surface was made using chemical etching technology. The effects of a nanotextured Ti surface on murine preosteoblastic cells’ proliferation, adherence, differentiation, and mineralization has been previously investigated. Therefore, nanophase metals seem to have great potential in both prosthetics and implantation, but more studies are required. Research revealed that nacre (the innermost layer of mollusk shells) powder promotes peri-implant osteogenesis in the tibias of domestic pigs. Micro-CT analysis and a histological study showed that this bioactive material promotes adequate bone formation around an implant surface. The possibility of nacre powder application in combination with surgical implant placement can be used as an alternative method to promote osseointegration [15,16,17].

Dentin matrix protein stimulates adhesion and proliferation and promotes human stem cell differentiation and mineralized matrix formation. Thus, a biologically modified Ti surface with dentine matrix protein is recommended to be applied to ensure better osseointegration in Ti implants [17].

As shown in the previous literature, the direct placement of dental implants with bone graft materials did not prevent peri-implant bone remodeling, and a reduction in bone thickness was still observed. Remarkably, the change in the buccal alveolar ridge was more noticeable. Consequently, this method can be used as an alternative to the existing treatment protocol for the placement of an implant in posterior areas [16].

We found that the guided implant surgical protocol is beneficial, because the final outcome can be based on a reliable treatment plan, reducing marginal bone resorption and improving implant longevity. The guided implant surgical (GIS) protocol is essential to avoid flap elevation and ensure maximum implant position while preserving marginal bone in the area around the implant. Bioactive glass can be then applied in grafting for optimal bone formation as a potential scaffold biomaterial. Further studies into GIS research will confirm these data and ensure customized, optimized treatment protocols for patients [18].

In terms of the mechanical response exhibited by bone tissue, a homogeneous strain is desired and can be affected by the different biomaterials used in the reconstruction of edentulous maxilla. The reduction in stress in zygomatic implants and prosthetic screws is achieved using zirconium, CoCr, and titanium, which are higher in strength and have better mechanical behavior than polymeric superstructures [19,20]. Obviously, further progress in prosthodontic technology largely depends on developments in materials science. Undoubtedly, the most significant contribution to fundamental scientific innovations and technological changes that are currently taking place in clinical prosthodontics is that of nanomaterials. Nanotechnology makes it possible to reduce the size of a material to the nanoscale, thus enhancing many of its properties, such as the surface hardness, the modulus of elasticity, the polymerization shrinkage, and the filler content, to improve the performance of well-known materials.

We very much hope that the information presented in this editorial will prove beneficial for future scientific research and technological innovations in the field of dentistry.
